# β-Glucan from *Lentinula edodes* prevents cognitive impairments in high-fat diet-induced obese mice: involvement of colon-brain axis

**DOI:** 10.1186/s12967-021-02724-6

**Published:** 2021-02-04

**Authors:** Wei Pan, Pengfei Jiang, Jinxiu Zhao, Hongli Shi, Peng Zhang, Xiaoying Yang, Joanna Biazik, Minmin Hu, Hui Hua, Xing Ge, Xu-Feng Huang, Yinghua Yu

**Affiliations:** 1 Jiangsu Key Laboratory of Immunity and Metabolism, Department of Pathogen Biology and Immunology, Xuzhou Medical University, Xuzhou, 221004 Jiangsu China; 2 Electron Microscope Unit, Mark Wainwright Analytical Centre, The University of New South Wales, Sydney, NSW 2052 Australia; 3 Illawarra Health and Medical Research Institute (IHMRI), School of Medicine, University of Wollongong, Wollongong, NSW 2522 Australia

**Keywords:** *Lentinula edodes*, β-Glucan, Cognition, Microbiota, Gut-brain axis, High fat diet, Obesity

## Abstract

**Background:**

Long-term high fat (HF) diet intake can cause neuroinflammation and cognitive decline through the gut-brain axis. (1, 3)/(1, 6)-β-glucan, an edible polysaccharide isolated from medical mushroom, *Lentinula edodes* (*L. edodes*), has the potential to remodel gut microbiota. However, the effects of *L. edodes* derived β-glucan against HF diet-induced neuroinflammation and cognitive decline remain unknown. This study aimed to evaluate the neuroprotective effect and mechanism of dietary *L edodes* β-glucan supplementation against the obesity-associated cognitive decline in mice fed by a HF diet.

**Methods:**

C57BL/6J male mice were fed with either a lab chow (LC), HF or HF with *L. edode*s β-glucan supplementation diets for 7 days (short-term) or 15 weeks (long-term). Cognitive behavior was examined; blood, cecum content, colon and brain were collected to evaluate metabolic parameters, endotoxin, gut microbiota, colon, and brain pathology.

**Results:**

We reported that short-term and long-term *L. edodes* β-glucan supplementation prevented the gut microbial composition shift induced by the HF diet. Long-term *L. edodes* β-glucan supplementation prevented the HF diet-induced recognition memory impairment assessed by behavioral tests (the temporal order memory, novel object recognition and Y-maze tests). In the prefrontal cortex and hippocampus, the β-glucan supplementation ameliorated the alteration of synaptic ultrastructure, neuroinflammation and brain-derived neurotrophic factor (BDNF) deficits induced by HF diet. Furthermore, the β-glucan supplementation increased the mucosal thickness, upregulated the expression of tight junction protein occludin, decreased the plasma LPS level, and inhibited the proinflammatory macrophage accumulation in the colon of mice fed by HF diet.

**Conclusions:**

This study revealed that *L. edodes* β-glucan prevents cognitive impairments induced by the HF diet, which may occur via colon-brain axis improvement. The finding suggested that dietary *L. edodes* β-glucan supplementation may be an effective nutritional strategy to prevent obesity-associated cognitive decline.

## Background

Obesity prevalence is increasing steadily around the world that has a devastating impact on the sustainable health of individuals in the long-term [1]. Obesity is not only associated with insulin resistance, type 2 diabetes mellitus, cardiovascular disease and steatohepatitis, but can also increases the risk of developing neurodegenerative diseases such as Alzheimer’s disease (AD) [2, 3]. Evidence shows that obesity and/or high fat feeding are associated with deficits in learning, memory, and executive functioning [4, 5] and potentially brain atrophy [6]. However, effective therapeutic manipulation still lacks. Thus, it is urgent to clarify the underlying mechanism of obesity-induced cognitive decline and develop a targeted therapeutic strategy.

Neuroinflammation is proposed to be an important pathophysiological hallmark underlying cognitive decline [7]. Microglia, being the resident immune cells of the central nervous system, plays an important role in maintaining brain homeostasis and contributes towards brain development under normal conditions. However, excessive microglial activation can mediate cognitive impairment via inducing progressive loss of neurons [8]. Hence, addressing neuroinflammation mediated by microglia bears great promise as a novel treatment strategy to reduce neuronal damage and to foster a permissive environment for further regeneration effort [9].

Gut microbiota can affect brain plasticity and cognitive function through the gut-brain axis. The gut microbiota maintains host intestinal homeostasis and regulates immunity. It is reported that *Bacteroides fragilis* of Bacteriodetes phylum can increase tight junction proteins expression and attenuate intestinal permeability, while *Ruminococcus* of Firmicutes phylum can degrade mucus [10]. In the mice fed by a long-term high-fat (HF) diet, an increase in intestinal permeability allows the translocation of bacteria or bacterial lipopolysaccharide (LPS) into the blood circulation and then induces systemic inflammation. Moreover, these cytokines can infiltrate in the brain via the blood–brain barrier and act on microglia to induce local production of proinflammatory cytokines, thereby triggering neuroinflammation and cognitive impairment. Furthermore, evidence from germ-free, antibiotic-treated and pathogen-free rodents has revealed gut microbiota dysbiosis negatively affect hippocampal neurogenesis and brain development via microglia activation [11, 12], suggesting a vital role of gut microbiota in cognitive function. Therefore, the gut-brain axis is considered as the potential therapeutic target for HF induced neuroinflammation and cognitive decline [13–16].

*Lentinula edodes* (*L. edodes*) is one of the most popular edible mushrooms in the global market, and (1, 3)/(1, 6)-β-glucan is its major bioactive component. The biological activities of the β-glucan have attracted more attention recently in the medical fields not only due to its nutritional value but also to the possible potential for therapeutic applications [17]. It has been reported that *L. edodes can* be used medicinally for diseases involving hyperlipidemia, hypertension and diabetes [17]. Interestingly, two studies have reported that the supplementation of *L. edodes* derived β-glucan for consecutive 28 days can improve gut microbiome dysbiosis in aged mice [18], and thereby improve insulin resistance in insulin-deficient type 2 diabetic rats [19]. Moreover, a recent cross-sectional study has shown that mushroom consumption had reduced the incidence rate of mild cognitive impairment in aged individuals in Singapore [20]. These findings suggest that the main ingredient of *L. edodes*, β-glucan, may have the potential to regulate gut microbiota and gut-brain axis. However, it is unknown if *L. edodes* derived β-glucan can prevent microbiota dysbiosis induced by HF diet and thereby improve cognitive impairments via gut-brain axis.

In this study, we designed short-term and long-term experiments of *L. edodes *derived β-glucan supplementation to examine acute effects on gut microbiota and chronic effects on cognition and its underlying gut-brain axis. The composition of gut microbiota was examined in a short-term experiment for 7 days prior to body weight alteration. Most research of gut microbiota studies in humans and rodents are chronic studies. For example, studies have shown that chronic HF diet induced microbial dysbiosis with regards to the diversity and composition [15, 21]. However, the study of gut microbiota after an acute HF diet and intervention is rare. The results of short-term experiment indicate that *L. edodes* derived β-glucan supplementation prevented the microbial dysbiosis which appears in the early stage of HF diet feeding, before the onset of significant weight gain and obesity. Furthermore, a similar gut microbiota composition was observed after long-term supplementation of the β-glucan. The chronic high fat diet-fed mice are a commonly used animal model of cognitive impairment reported in the literature. In the long-term experiment, we assessed the effects of dietary *L. edodes* β-glucan supplementation by measuring behavioral tests, synaptic ultrastructure, neuroinflammation and brain-derived neurotrophic factor (BDNF) expression in the critical brain region, prefrontal cortex and hippocampus. Moreover, the intestinal parameters, including the colonic mucus thickness and tight junction protein expression, as well as serum endotoxin (LPS) level, were also evaluated. We demonstrated that chronic *L. edodes* derived β-glucan supplementation prevented HF diet-induced cognitive deficits with improvement in the gut-brain axis.

## Materials and methods

### Animals and treatment

Sixty C57Bl/6J male mice (9 weeks old) were purchased from the Experimental Animal Center of Xuzhou Medical University (Xuzhou, China, SCXK (Su) 2015–0009), and all mice were housed in SPF conditions (temperature 22 °C, 12 h light/dark cycle) and given free access to standard food and water. The mice were acclimatized for 7 days before the experiment and were randomly divided into two experiments: short-term experiment and long-term experiment (N = 30 per experiment). For each experiment, mice were further assigned to three groups (N = 10 per group): (I) Mice were fed a lab chow (LC) diet (5% fat by weight) as a control (LC) group. The LC diet (lab autoclavable rodent diet for maintenance, Beijing Keao Xieli Feed Co., LTD) is grain-based including, ground corn, dehulled soybean meal, fish powder, ground wheat, brewers dried yeast, soybean oil, salt, multivitamin and minerals et al.; (II) Mice received the HF diet (30% fat by weight) as the HF group. HF diet was made from semi-synthetic materials according to the recommendation of “AIN93 Diet for Laboratory Rodents”. The detailed composition contains lard 260 g, soybean oil 55 g, cornstarch 193 g, sucrose 192 g, gelatine 50 g, casein 130 g, methionine 3 g, cellulose 51 g, minerals 50 g and vitamins 13 g; and (III) Mice were fed the HF diet supplemented with the β-glucan from *L. edodes* (500 mg/Kg food, β-glucan at the dose of ~ 1.5 mg per mouse/day, ~ 60 mg /Kg body weight) [22, 23] as the HFL group. The β-glucan from *L. edodes* (≥ 98%, Yuanye Biotechnology Co., Ltd, Shanghai, China) was added and mixed into the HF diet. For the short-term experiment, mice received the respective diets for 7 days. Body weight and food intake were recorded every day. Mice were then euthanized, their cecal contents were collected and stored at − 80 °C for further analyses. For long-term experiments, mice were administered the three diets for 15 weeks. Body weight and food intake were measured on the last day of each week. The cognitive behaviour tests were performed (N = 10 per group), including the temporal order memory test, the novel object recognition test and the Y-maze test. Mice were sacrificed 4 days after behavioural testing with CO_2_. Liver and fat pads (subcutaneous, epididymal and brown) were dissected and weighed. Blood serum, intestinal, liver and brain tissues were also collected and stored in − 80 °C for further analyses. All mice in the experiment were fed ad libitum. All animal care and experiments were approved and carried out according to the ethics committee of Xuzhou Medical University.

### Behavioral testing

The temporal order memory test was performed based on methods previously described [15]. Briefly, the experiment comprised two sample trials and one test trial with an inter-trial interval of 60 min between each trial. Place mice in behavioral testing room 1 h before the test so they can acclimatize to the conditions. In each sample trial, the mice were allowed to explore two copies of the same object for 4 min; the objects were different between the two sample trials (sample trial 1: object A and A’; sample trial 2: object B and B’). During the test trial, one object from sample trial 1 (A; old familiar) and another object from sample trial 2 (B; recent familiar) were presented parallel and mice were allowed to explore the open field undisturbed for 3 min. A discrimination ratio was calculated by using the formula [(old familiar time − recent familiar time)/total exploration time]. Intact object recognition memory for temporal order was considered if the mice spent more time exploring the old familiar object compared with the recent familiar object.

The novel object recognition test (ORT) was performed based on methods previously described [24]. Briefly, there are three stages in the ORT. The first stage is habituation, in which a mouse is allowed to explore the open field for 5 min. After 24 h, beginning the training stage, in which the mouse allowed to explore the arena for 5 min with 2 identical objects placed parallel. After 1 h, retention session takes place. Mice are allowed to explore the arena with one of the familiar objects and one novel object placed parallel for 5 min. The discrimination index was evaluated by using the formula [Time with recent object/(Time with the older object + Time with recent object)] × 100.

The Y-maze test was performed based on methods previously described [25]. After acclimatization of the mice, label the arms of the maze with different pictures. Put the mouse in the center and allow to explore the maze undisturbed for 8 min. Record the number of all arm entries and alternations. An alternation is defined as the mouse entering all three arms consecutively. The alternation triplet (%) was calculated as [number of successful alternations/(total number of arms entries-2) × 100].

### Intraperitoneal glucose tolerance test (GTT)

Glucose tolerance test was performed 3 days after behavioral testing. After fasting for 16 h, mice were given a glucose solution in the abdominal cavity at a dose of 2 g/kg. The tail-tip blood samples were collected at 0, 30, 60, 90, and 120 min after injection, and a glucose monitor was used to detect the concentration of blood glucose. The curve of blood glucose over time was drawn by Graphpad 8 software and the total area under the curve (AUC) was calculated.

### Transmission electron microscopy (TEM)

After transcardial perfusion with saline, brain tissues were taken out and 1 mm^3^ of tissue blocks from the cornus ammonis (CA1), CA3 and dentate gyrus (DG) regions of the hippocampus and prefrontal cortex were dissected. Samples were fixed in a 2% paraformaldehyde-2.5% glutaraldehyde mixture for 24 h and treated post-fixation with 1% osmium tetroxide (OsO4) for 2 h, before dehydration in an ascending graded ethanol series and embedding in epoxy resin. Sections (70 nm) were cut and stained with 4% uranyl acetate and 0.5% lead citrate. Ultrastructure of synapses was measured under a transmission electron microscope (FEI, Portland, USA), and synaptic morphometrics were studied. Three indexes (Postsynaptic density, synaptic clefts width and the curvature of the synaptic interface) were compared using Image J software as described previously [26].

### LPS determination

LPS levels in sera were detected using a chromogenic end-point TAL kit (Xiamen Bioendo Technology Co., Ltd, Xiamen, China) according to the manufacturer’s protocol. The absorbance was determined at a wavelength of 545 nm using a spectrophotometer (Asuragen ClinBio128, USA). All samples for LPS measurements were performed in duplicate.

### Cecal content collection

Fresh cecal contents of mice after short-term and long-term experiments were collected into individual sterile EP tubes, quickly frozen in liquid nitrogen, and then transferred into an – 80 ℃ cryogenic freezer until DNA extraction for microbiota analysis by 16S rRNA gene sequencing.

### Gut microbiota analysis

Genomic DNA amplification, operational taxonomic units (OTUs) and sequencing were conducted as previously reported [14, 15]. Briefly, the DNA from cecal contents in short-term and long-term experiments were extracted following the instruction of HiPure stool DNA kit (Magen, Beijing, China). The 16S rDNA V3-V4 region of the Eukaryotic ribosomal RNA gene were amplified by PCR using primers 341F: CCTACGGGNGGCWGCAG; 806R: GGACTACHVGGGTATCTAAT, where the barcode is an eight-base sequence unique to each sample using the MiSeq Illumina 2500 platform (Shanghai Majorbio Biopharm Technology Co., Ltd., Shanghai, China) following the standard protocols. The 16S rRNA sequencing data set generated by MiSeq operation was merged and decomposed into each sample using the QIIME version 1.9.0. OTUs were clustered with 97% similarity cutoff using UPARSE (version 7.1 http://drive5.com/uparse/), and chimeric sequences were identified and removed using UCHIME. The taxonomy of each 16S rRNA gene sequence was analyzed by RDP Classifier (http://rdp.cme.msu.edu/) against the silva (SSU115)16S rRNA database using confidence threshold of 70%. OTUs with number less than 0.005% of the total sequence number were excluded. The linear discriminant analysis (LDA) effect size (LEfSe) was used to detect the features of gut microbiota with significant difference abundances between designated taxa with the Kruskal−Wallis rank-sum test.

### Histological staining and immunohistochemistry

All animals were killed using CO_2_ asphyxiation followed by cervical dislocation. The gastrointestinal tracts were quickly removed. The colons were gently separated, by cutting at the cecum-colon junction and the rectum, and immediately preserved in Carnoy’s fixative (fresh anhydrous methanol:chloroform:glacial acetic acid in the ratio 60:30:10). The descending colons were fixed in Carnoy’s solution (twice for 3 h). The colons were then washed in anhydrous methanol for 2 h, placed in cassettes and stored in anhydrous methanol at 4 ℃ for further use. For the detection of colonic mucus layer thickness, post Carnoy’s fixation, methanol-stored colon samples were embedded in paraffin, cut into thin Section (5 μm), and mounted on glass slides with Alcian blue staining as previously described [27], and the thickness of colonic mucus layer was measured using Image J.

The immunohistochemical staining has been described in our previous study [15]. Briefly, fixed colon tissues were embedded in paraffin and sectioned at 5 μm. The sections were rehydrated in xylene and then in graded ethanol solutions. The sections were then washed in 3% H_2_O_2_ in methanol for 30 min. For brain tissues, fixed tissues were sectioned at 20 μm, using phosphate buffer saline (PBS) washed 3 times for 10 min, and then washed in 1% H_2_O_2_ in PBS for 30 min. All sections were blocked with 5% normal goat serum and incubated with indicated primary antibodies at 4 °C overnight. Primary antibodies were anti-F4/80 (ab16911, Abcam, UK, 1: 1000 dilution) for the colon and anti-Iba1 (019-19741, Wako Pure Chemical Industries, Japan, 1: 1000 dilution) for the brain. Following primary antibody incubation, sections were washed with PBS and then incubated with goat anti-rabbit IgG H&L (ab6702, Abcam, UK, 1: 500 dilution) at 37 °C for 2 h. Finally, using the DAB peroxidase substrate kit (Cell Signaling Technology, Boston, USA) to wash the sections and the sections were counterstained with hematoxylin (Sigma-Aldrich, St. Louis, USA). Six fields from three sections of each mouse were viewed by OLYMPUS IX51 microscope (Tokyo, Japan) and digital photographs were captured. Image J software was used to quantify the cells of F4/80 and Iba1 immunoreactivity on each field. The immunohistochemical staining F4/80 and Iba1 were quantified as a percentage of positive cells on each field.

### Western blotting

Western blot assay was performed as described previously [28]. Briefly, proteins were extracted from tissues of the colon, hippocampus and prefrontal cortex in cell lysis buffer containing RIPA buffer (Sigma-Aldrich, St. Louis, USA), Protease Inhibitor Cocktail (Sigma-Aldrich, St. Louis, USA) and 1 mM PMSF (Sigma-Aldrich, St. Louis, USA), and then quantified by BCA assay (Beyotime Biotech, Beijing, China). 20–40 μg of protein, was separated by electrophoresis in 10% SDS-PAGE and separated proteins were transferred to polyvinylidene difluoride (PVDF) membranes with a Bio-Rad electrophoresis system (Hercules, CA, USA). The membranes were blocked with 5% skim milk powder and then incubated overnight at 4 °C with different primary antibodies diluent (Occludin, ab167161, Abcam, UK, 1: 2000 dilution; BDNF, ANT-010, Alomone Labs, Israel, 1:200 dilution; PSD-95, #3450, Cell Signaling Technology, Boston, USA, 1: 1000 dilution; GAPDH, A2077, ABclonal Biotechnology Co., Ltd, USA, 1:2000 dilution). The membranes were then washed three times for 10 min and incubated with the anti-rabbit IgG conjugated with horseradish peroxidase (sc-2030, Santa Cruz Biotechnology, USA, 1:2000 dilution)) for 1 h at room temperature. Immunodetection was performed using Clarity™ ECL western blot substrate (Bio-Rad, USA) and visualized with the ChemiDoc Touch imaging system (Bio-Rad, USA). The expression of protein in each sample was normalized to GAPDH.

### RNA extraction and quantitative real-time PCR (RT-qPCR)

RNA extraction and RT-qPCR of colon, hippocampus and prefrontal cortex were performed based on methods previously described [29]. Briefly, total RNA was extracted with TRIzol (Thermo Fisher Scientific, USA) from tissues of the colon, hippocampus and prefrontal cortex. RNA quantity was measured at 260 nm and purity was assessed by the optical density 260 nm/optical density 280 nm ratio. Then, 1 μg RNA for each sample was reverse-transcripted to cDNA using a high-capacity cDNA reverse transcription kit (Takara, Japan). qPCR was performed using the SYBR GREEN Master Mix (TaKaRa, Japan) and determined on a real-time PCR detection system (Bio-Rad, USA). Results were calculated using the comparative CT method (2^−ΔΔCt^) and expressed relative to the expression of the housekeeping gene GAPDH. Primer sequences were as follows: TNF-α-forward(F): 5′-CTTGTTGCCTCCTCTTTTGCTTA-3′, TNF-α-reverse(R): 5′-CTTTATTTCTCTCAATGACCCGTAG-3′; IL-1β-forward(F): 5′-TGGGAAACAACAGTGGTCAGG-3′, IL-1β-reverse(R): 5′-CTGCTCATTCACGAAAAGGGA-3′; IL-6-forward(F): 5′-TCACAGAAGGAGTGGCTAAGGACC-3′, IL-6-reverse(R): 5′-ACGCACTAGGTTTGCCGAGTAGAT-3′; β-GAPDH-forward(F): 5′-AGAAGGTGGTGAAGCAGGCATC-3′, β-GAPDH-reverse(R): 5′-CGAAGGTGGAAGAGTGGGAGTTG-3’.

### Statistical analysis

All data are reported as mean ± standard error of the mean (SEM). Data analysis was performed using one-way analysis of variance (ANOVA), followed by Tukey post-hoc test. Statistical analyses were performed in SPSS 20.0. *P* < 0.05 was considered statistically significant.

## Results

### Short-term *L. edodes* derived β-glucan supplementation attenuated the gut microbiota dysbiosis induced by HF diet

**Fig. 1:**
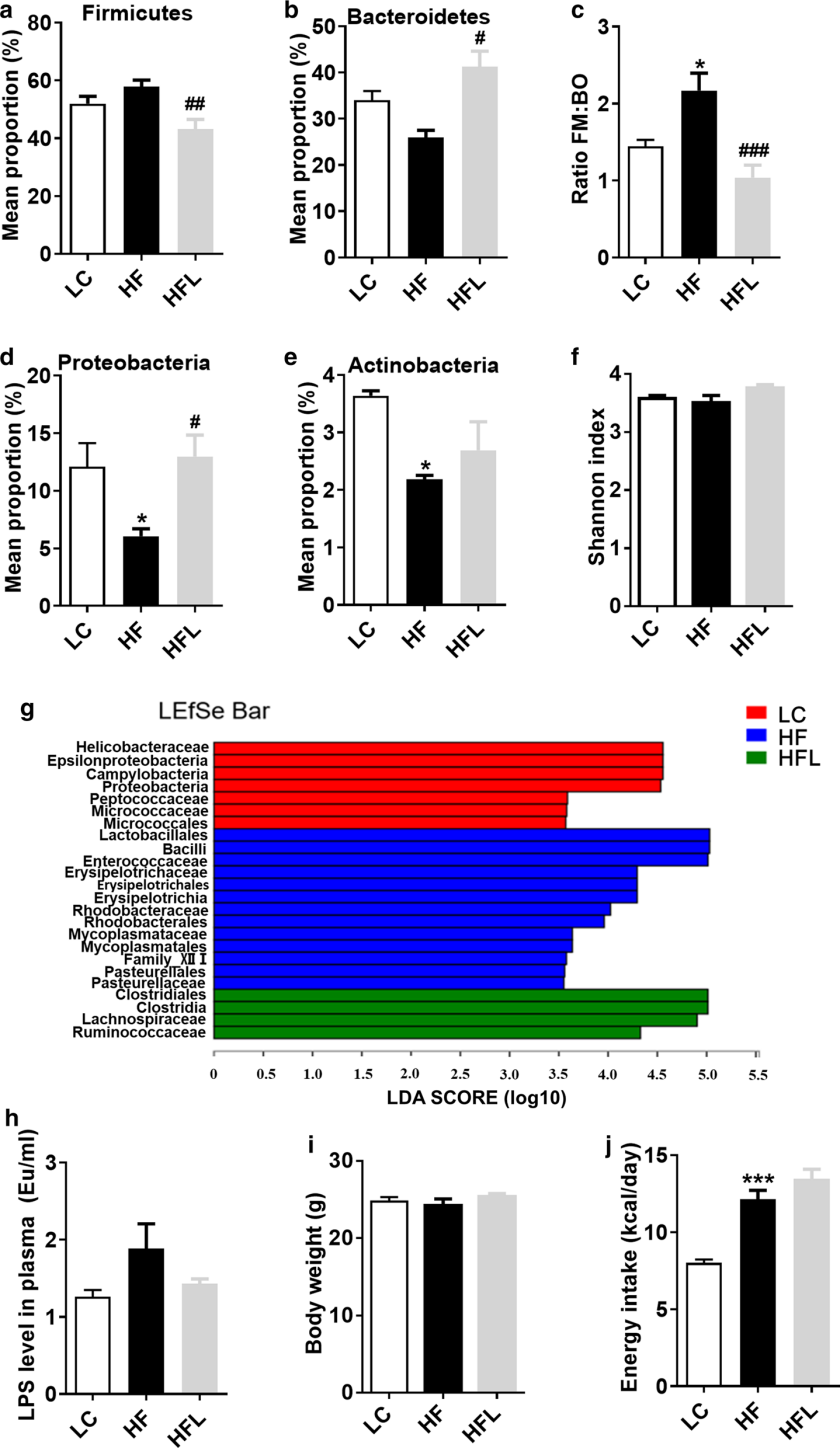
Short-term *L. edodes* derived β-glucan supplementation attenuated the gut microbiota dysbiosis induced by HF diet. **a** The relative abundance of Firmicutes, **b** the relative abundance of Bacteroidetes, **c** the ratio of Firmicutes (FM) to Bacteroidetes (BO), **d** the relative abundance of Proteobacteria, **e** the relative abundance of Actinobacteria, **f** the Shannon index, **g** the linear discriminant analysis (LDA) effect size results on mice gut microbiomes, **h** the plasma LPS levels in the sera, **i** body weight, **j** energy intake. Data are presented as mean ± standard error of means (SEM). *n* = 6 per group. ^*^P < 0.05, ^***^P < 0.001, vs lab chow diet group (LC); ^#^*P* < 0.05, ^##^ *P* < 0.01, ^###^*P* < 0.001, vs high-fat diet group (HF). HFL, *L. edodes* β-glucan supplementation in HF diet group

Our previous studies have shown that chronic HF diet led to microbial dysbiosis with regards to diversity and composition [15, 16]. It is unknown if a short-term HF diet could alter microbiota and *L. edodes* derived β-glucan could prevent HF diet-induced microbiota shift. The profiles of gut microbiota after a short-term HF diet with or without *L. edodes* derived *β-*glucan supplementation for 7 days by 16S rRNA sequencing. Only a slight increase in mean proportion of Firmicutes, and decrease in mean proportion of Bacteroidetes were observed in short-term HF diet-fed mice compared to LC mice. However, the ratio of Firmicutes to Bacteroidetes were significantly elevated in the HF group (Fig. 1a–c). Nevertheless, *L. edodes* β-glucan supplementation prevented the alterations mentioned above (Fig. 1a–c). Meanwhile, the mean proportion of Proteobacteria and Actinobacteria was significantly decreased by the HF diet, while short-term *L. edodes* supplementation improved the abundance of Proteobacteria (*P* < 0.05, Fig. 1d, e). While there was no significant difference in bacterial diversity (in Shannon index) among the three groups (Fig. 1f).

Using a linear discriminant analysis (LDA) effect size (LEfSe) calculation, 24 abundant taxonomic classes with LDA score higher than 3.0 (Fig. 1g) were identified. In detail, bacteria belonging to order Lactobacillales, class Bacilli, family Enterococcaceae, family Erysipelotrichaceae, order Erysipelotrichales, class Erysipelotrichia, family Rnodobacteraceae, order Rhodobacterales, family Mycoplasmataceae, order Mycoplasmatales, family Family_Xlll, order Pasteurellales and family Pasteurellaceae were elevated significantly in HF mice compared to control. Furthermore, *L. edodes* β-glucan supplementation significantly increased the order Clostridiales, class Clostridia, family Lachnospiraceae and family Ruminococcaceae (Fig. 1g). Consistent with the altered microbiota, slightly elevated plasma levels of LPS were observed in HF diet-fed mice, which was reversed by the supplementation of *L. edodes* β-glucan (Fig. 1h). Together, these results indicated that short-term *L. edodes* β-glucan attenuated the gut microbiota dysbiosis induced by HF diet.

Furthermore, the short-term HF diet administration increased the energy intake, but did not increase the body weight (Fig. 1i, j). The short-term *L. edodes* β-glucan supplementation did not affect the body weight compared to control mice and HF mice (Fig. 1i). Moreover, the β-glucan supplementation increased energy intake compared to control, but not compared to HF mice (Fig. 1j).

### Long-term *L. edodes* derived β-glucan supplementation prevented the gut microbiota dysbiosis induced by HF diet

In long-term HF diet-fed mice, a significant increase in the mean proportion of Firmicutes (Additional file 1: Fig. S1A, B) and Proteobacteria and a significant decline of Bacteroidetes were observed compared to LC mice (Additional file 1: Fig. S1D). Moreover, the ratio of Firmicutes to Bacteroidetes was significantly elevated in the HF group (Additional file 1: Fig. S1C). Importantly, *L. edodes* β-glucan supplementation prevented the alterations mentioned above, except for the mean proportion of Bacteroidetes (Additional file 1: Fig. S1A–D). In addition, *L. edodes* β-glucan supplementation significantly increased the mean proportion of Actinobacteria in HF diet-fed mice, although the HF diet did not obviously affect the phylum compared to the LC diet (Additional file 1: Fig. S1E). However, there was no significant difference in bacterial diversity (in Shannon index) among the three groups (Additional file 1: Fig. S1F), which was similar to the results in the short-term administration of *L. edodes* β-glucan. Taken together, these results indicated that long-term *L. edodes* β-glucan also prevented the gut microbiota dysbiosis induced by HF diet.

### Long-term *L. edodes* derived β-glucan supplementation prevented HF diet-induced cognitive decline

**Fig. 2:**
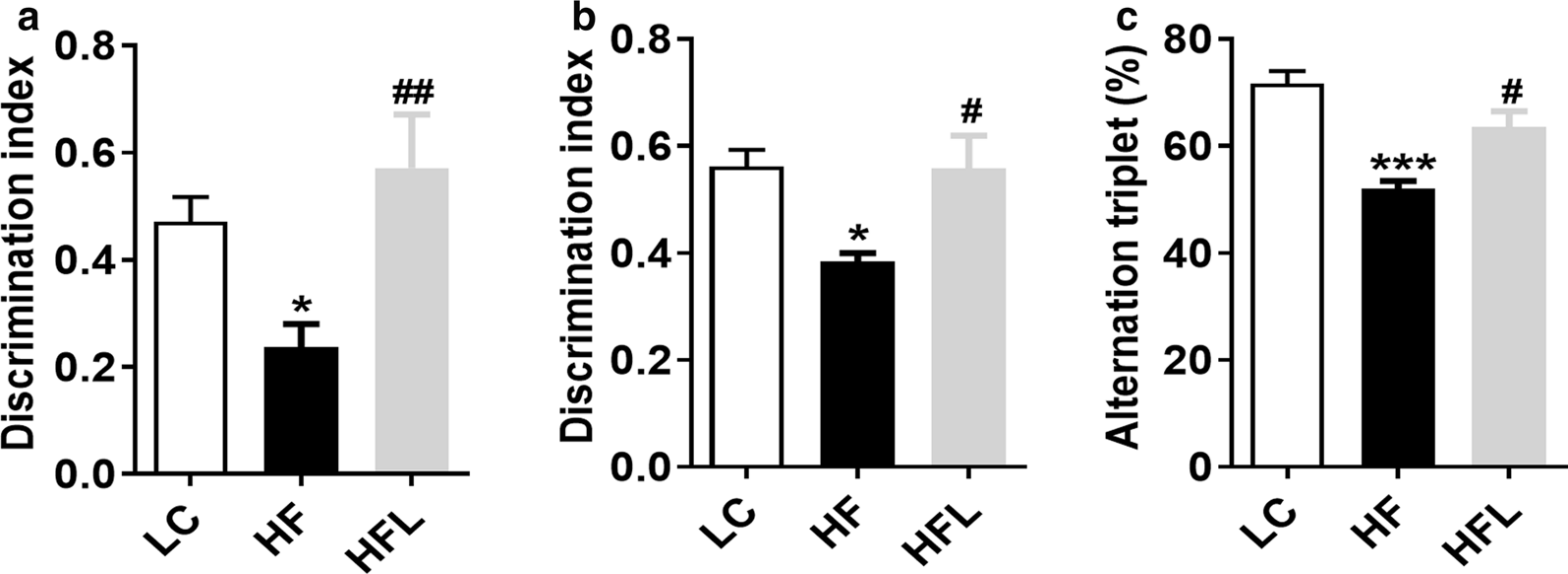
Long-term *L. edodes* derived β-glucan supplementation prevented HF diet-induced cognitive decline. **a** Discrimination index in the temporal order memory test, **b** discrimination index in the novel object recognition test, **c** proportion of correct alternations in the Y-maze test. *n* = 10 per group. Data are presented as mean ± SEM. ^*^P < 0.05, ^**^P < 0.01, ^***^P < 0.001, vs lab chow diet group (LC); ^#^*P* < 0.05, ^##^*P* < 0.01, vs high-fat diet group (HF). HFL, *L. edodes* β-glucan supplementation in HF diet group

Since that gut microbiota dysbiosis and obesity are strongly linked to cognitive decline [16], we further determined whether *L. edodes* β-glucan could prevent cognitive decline induced by chronic HF diet. In the temporal order memory test, chronic HF diet resulted in a decrease in recognition memory compared with the LC mice (*P* < 0.05, Fig. 2a). Compared with the LC group, the HF group exhibited a decreased discrimination index, suggesting that the recognition memory of HF mice was impaired. However, *L. edodes* β-glucan administration significantly increased the discrimination index, which even exceeded the LC mice (*P* < 0.001, Fig. 2a). Moreover, a similar protective effect of *L. edodes* β-glucan supplementation was observed in the novel object recognition test (*P* < 0.05, Fig. 2b). In addition, the alternation triplet of HF mice was lower than that of LC and *L. edodes* β-glucan fed mice in the Y-maze test (*P* < 0.05, Fig. 2c). All these behavior results implied that *L. edodes* β-glucan prevents HF diet-induced cognitive decline. Furthermore, the cumulative body weight gain and final body weight of HF mice were significantly higher than those of LC mice, and chronic *L. edodes* β-glucan significantly lowered body weight gain and final body weight in HF mice (*P* < 0.01, Additional file 2: Fig. S2A, B). However, there was no obvious change of cumulative energy intake in the mice fed by *L. edodes* β-glucan plus HF diet in comparison to HF mice (*P* < 0.05, Additional file 2: Fig. S2C). In line with the body weight change, the mass of liver, subcutaneous fat, epididymal fat and brown fat was significantly lower in HF mice following *L. edodes* β-glucan supplementation (all *P* < 0.05, Additional file 2: Fig. S2D, E). In addition, *L. edodes* β-glucan adminstration markedly improved glucose intolerance (*P* < 0.05, Additional file 2: Fig. S2F). Together, these results suggested *L. edodes* β-glucan prevented HF-induced obesity and cognitive decline.

### Long-term *L. edodes* derived β-glucan supplementation maintained colonic integrity and alleviated inflammation induced by HF diet

**Fig. 3:**
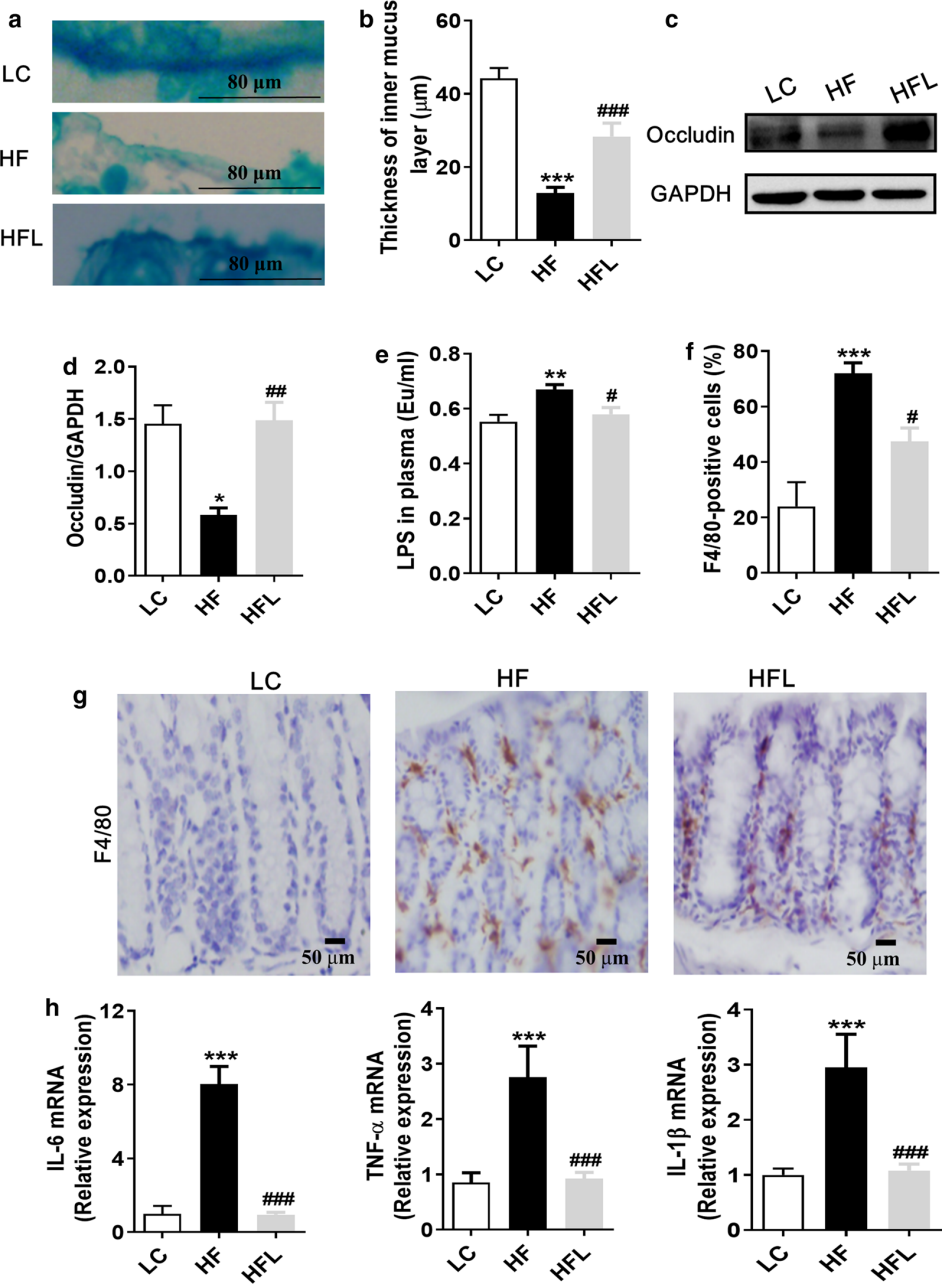
Long-term *L. edodes* derived β-glucan supplementation maintained colonic integrity and alleviated inflammation induced by HF diet. **a** Representative figure of alcian blue staining for colon, **b** quantification of colonic mucus layer thickness, **c** protein expression levels of occludin in the colon, **d** plasma LPS levels, **e** the representative image of immunohistochemical staining of colonic F4/80-positive cells, **f** quantification of colonic F4/80-positive cells, **g** mRNA expression of TNF-α, IL-6 and IL-1β in the colon*.* Data are presented as mean ± SEM. *n* = 3 per group. ^*^P < 0.05, ^**^P < 0.01, ^***^P < 0.001, vs lab chow diet group (LC); ^#^*P* < 0.05, ^##^*P* < 0.01, ^###^*P* < 0.001, vs high-fat diet group (HF). HFL, *L. edodes* β-glucan supplementation in HF diet group

Gut microbiota dysbiosis is positively associated with the damage of the intestinal barrier and endotoxinemia [16]. Therefore the colonic mucus and epithelium tight junction proteins were examined in response to *L. edodes* β-glucan supplementation. With Alcian blue staining, we found that the mucosal thickness in the colon was decreased in HF diet-fed mice compared to the LC group, while *L. edodes* β-glucan supplementation increased colonic mucosal thickness (*P* < 0.001, Fig. 3a, b). Moreover, western blot results showed that the protein level of occludin in the colon of HF diet mice was significantly decreased, while *L. edodes* β-glucan supplementation upregulated the protein expression of occludin (*P* < 0.01, Fig. 3c, d), indicative of enhanced tight junctions of colonic epithelial tissues. Consistently, the level of serum LPS was significantly increased with a chronic HF diet, which was reduced with *L. edodes* β-glucan supplementation compared to the HF group (*P* < 0.05, Fig. 3e). Thus, these results suggested that chronic *L. edodes* β-glucan supplementation maintained colonic integrity in the context of the HF diet.

The macrophage is one of the most abundant immune cells in the colon [30], which plays a vital role in intestinal immunity and homeostasis [31]. The immunohistochemical staining showed that compared with the LC group, the number of macrophage marker F4/80 positive cells in the HF group were significantly increased (*P* < 0.001, Fig. 3f, g); however, the number of F4/80-positive cells was markedly reduced by *L. edodes* β-glucan supplementation (*P* < 0.05, Fig. 3f, g), suggesting inhibition of macrophage activation. Consistent with the immunohistochemical staining results, RT-PCR results showed that the levels of proinflammatory cytokines IL-6, TNF-α and IL-1β were up-regulated in the colon of HF mice (*P* < 0.001, Fig. 3h), while *L. edodes* β-glucan inhibited the expression of these proinflammatory cytokines induced by HF diet (*P* < 0.01, Fig. 3h). Overall, these results suggested that the *L. edodes* derived β-glucan alleviated HF diet-induced colonic inflammation.

### Long-term *L. edodes* derived β-glucan supplementation inhibited the microgliosis and neuroinflammation in the mice fed HF diet

**Fig. 4:**
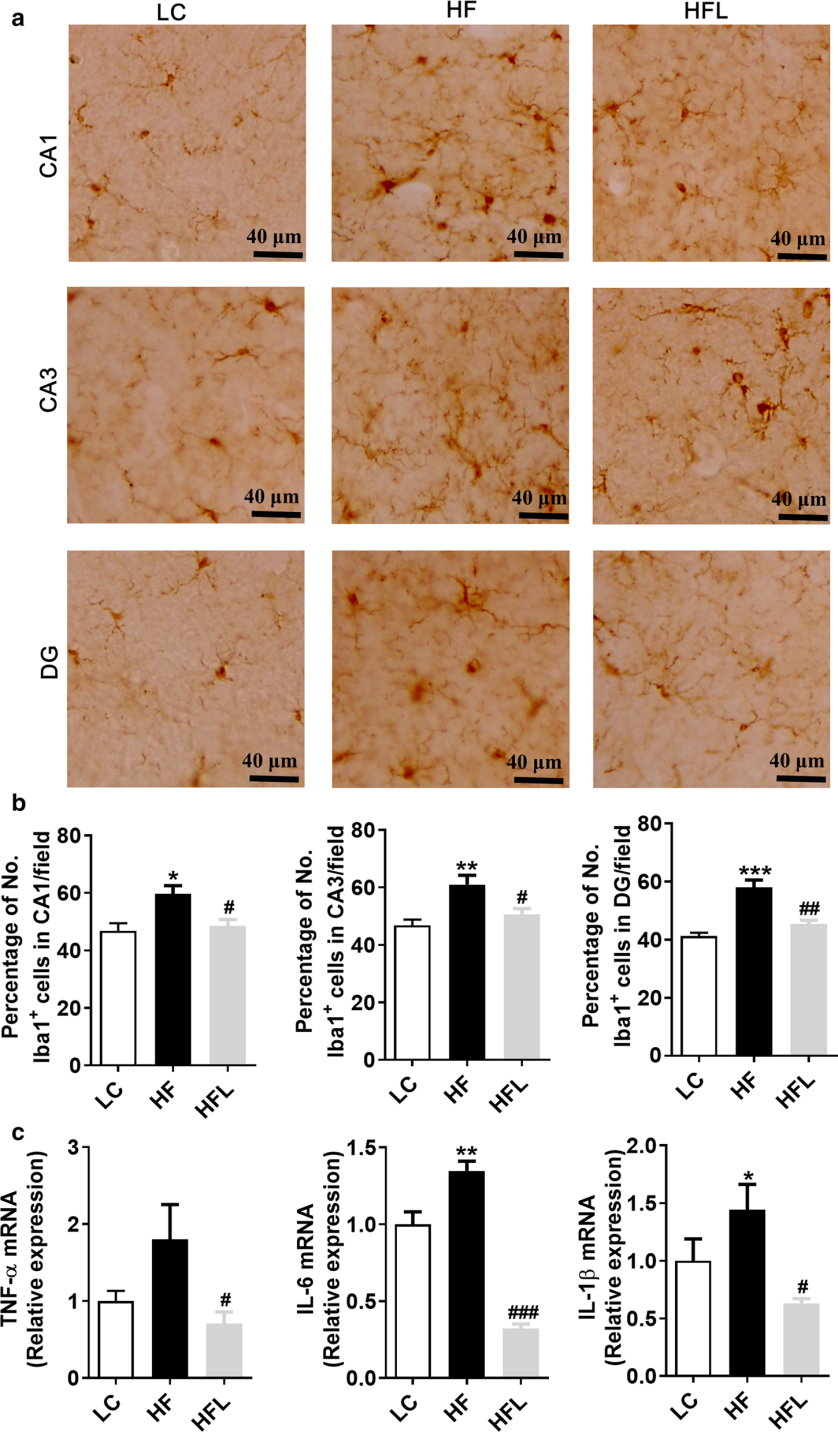
Long-term *L. edodes* derived β-glucan supplementation inhibited the microgliosis and neuroinflammation in the hippocampus of mice fed HF diet. **a** The representative image of immunohistochemistry staining of Iba1 in the cornus ammonis (CA1), CA3 and dentate gyrus (DG) regions of hippocampus, **b** the statistical results of Iba1^+^ positive cells in mentioned regions. **c** mRNA expression of TNF-α, IL-6 and IL-1β in the hippocampus. Data are presented as mean ± SEM. *n* = 3 per group. ^*^P < 0.05, ^**^P < 0.01, ^***^P < 0.001, vs lab chow diet group (LC); ^#^*P* < 0.05, ^##^*P* < 0.01, ^###^*P* < 0.001, vs high-fat diet group (HF). HFL, *L. edodes* β-glucan supplementation in HF diet group

**Fig. 5:**
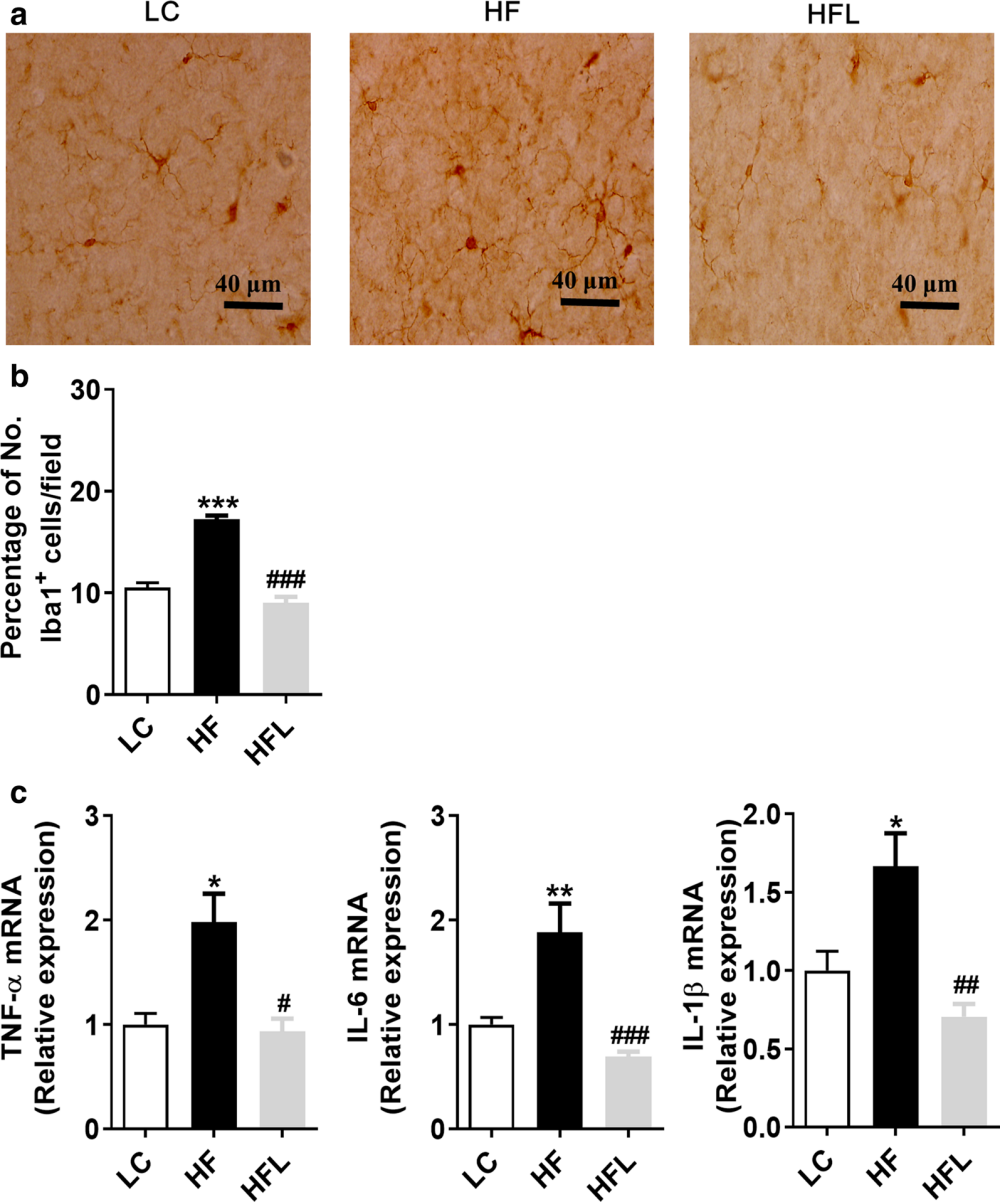
Long-term *L. edodes* derived β-glucan supplementation inhibited the microgliosis and neuroinflammation in the prefrontal cortex of mice fed HF diet. **a** The representative image of immunohistochemistry staining of Iba1 in PFC, **b** the statistical results of Iba1^+^ positive cells in PFC. (C) mRNA expression of TNF-α, IL-6, and IL-1β in the PFC. Data are presented as mean ± SEM. *n* = 3 per group. ^*^P < 0.05, ^**^P < 0.01, vs lab chow diet group (LC); ^#^*P* < 0.05, ^##^*P* < 0.01, ^###^ *P* < 0.001, vs high-fat diet group (HF). HFL, *L. edodes* β-glucan supplementation in HF diet group

It is reported that intestinal barrier impairment and endotoxinemia can mediate microglial activation and neuroinflammation through the gut-brain axis [16]. Moreover, the hippocampus and the cortex, regions implicated in cognitive processing, learning and memory, may be particularly vulnerable to inflammation in obesity [32, 33]. The present study further determined the effects of *L. edodes* on microgliosis and neuroinflammation induced by the HF diet in the hippocampus and prefrontal cortex (PFC). Using Iba1 as the marker of microglia, we observed that the HF diet increased microglia number in the hippocampal regions, including cornus ammonis (CA1), CA3 and dentate gyrus (DG), while *L. edodes* β-glucan significantly reduced microglial number (all *P* < 0.05, Fig. 4a, b). Moreover, the downregulated expression of proinflammatory cytokines (TNF-α, IL-6, IL-1β) was observed in the hippocampus after *L. edodes* β-glucan supplementation (all *P* < 0.05, Fig. 4c). In the PFC, *L. edodes* β-glucan could inhibit the proliferation of microglia (*P* < 0.01, Fig. 5b) and downregulate the expression of proinflammatory cytokines (TNF-α, IL-6, IL-1β) induced by the HF diet (all *P* < 0.05, Fig. 5c). These results indicated that *L. edodes* β-glucan inhibited the microgliosis and inflammation in the hippocampus and PFC of mice fed the HF diet.

### Long-term *L. edodes* derived β-glucan supplementation alleviated the synaptic impairment in the mice fed HF diet

**Fig. 6:**
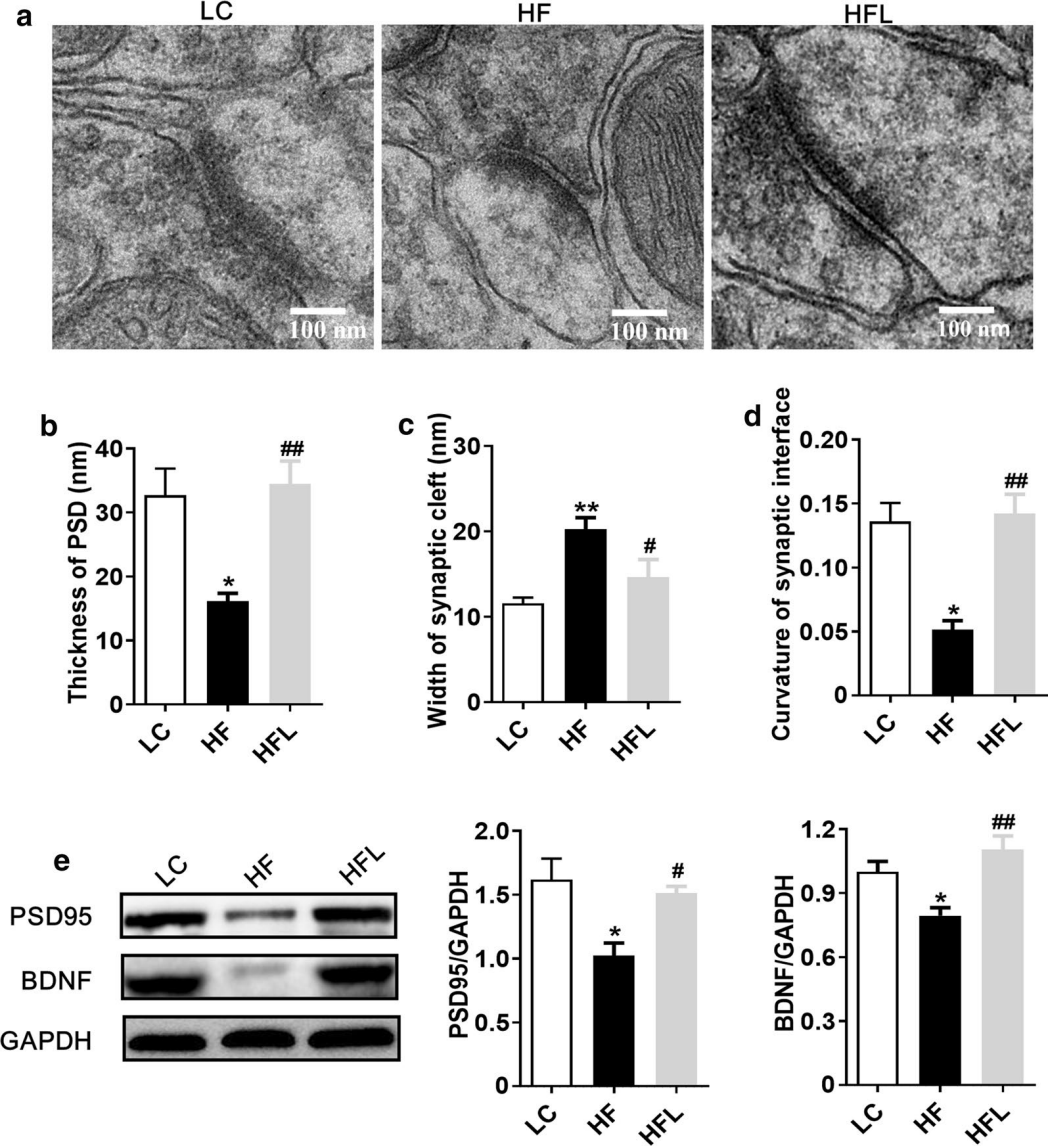
Long-term *L. edodes* derived β-glucan supplementation alleviated the synaptic impairment in the hippocampus of mice fed HF diet. **a** Representative electron micrograph of synaptic ultrastructure in the hippocampus cornus ammonis (CA1), CA3 and dentate gyrus (DG) regions, **b** statistical analysis of the thickness of PSD, the width of synaptic cleft (**c**), and curvature of the synaptic interface in the hippocampus (**d**), **e** protein expression levels of BDNF and PSD-95 in the hippocampus. Data are presented as mean ± SEM. *n* = 3 per group. ^*^P < 0.05, ^**^P < 0.01, vs lab chow diet group (LC); ^#^*P* < 0.05, ^##^*P* < 0.01, vs high-fat diet group (HF). HFL, *L. edodes* β-glucan supplementation in HF diet group

**Fig. 7:**
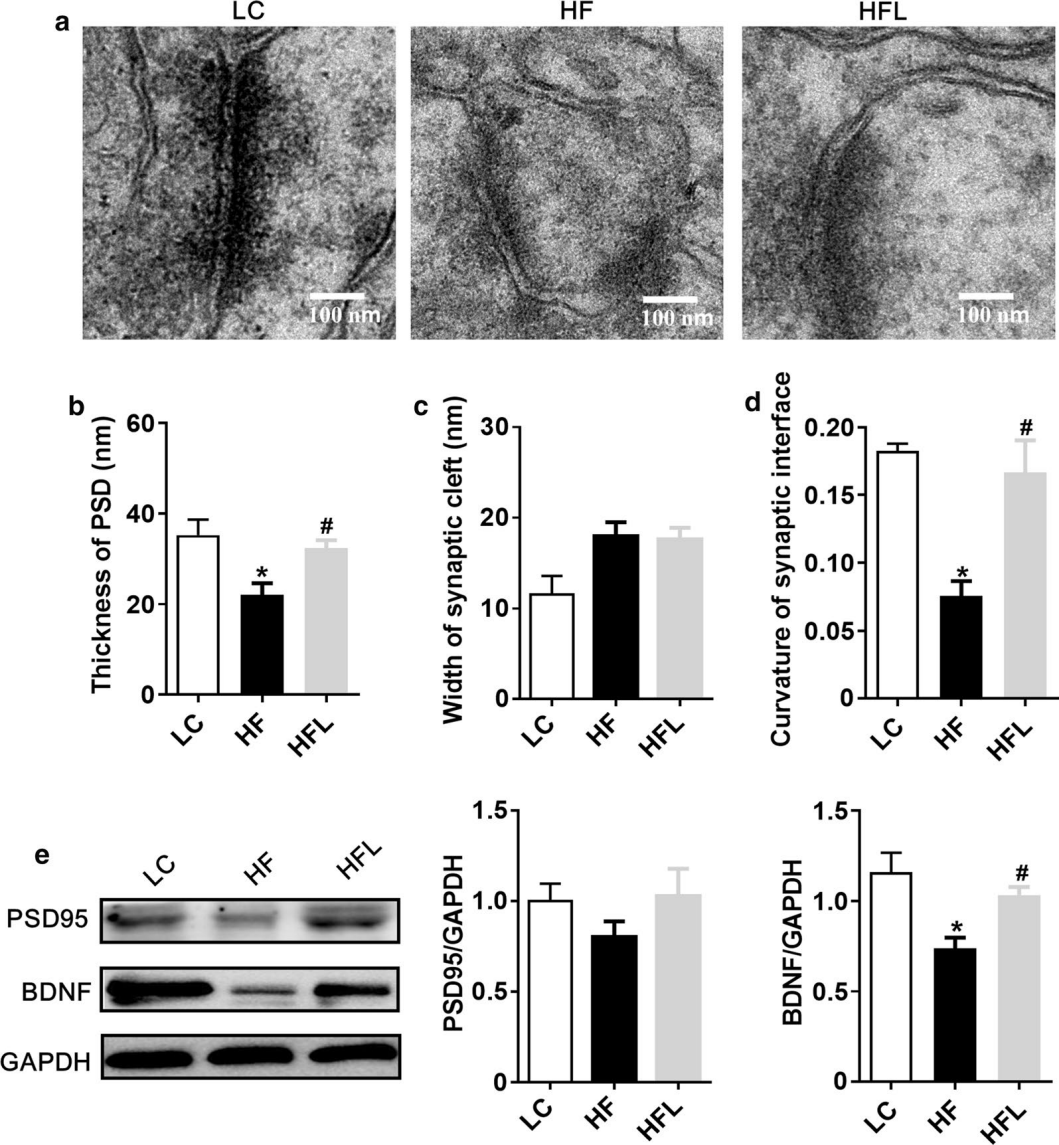
Long-term *L. edodes* derived β-glucan supplementation alleviated the synaptic impairment in the prefrontal cortex of mice fed HF diet. **a** Representative electron micrograph of synaptic ultrastructure in the prefrontal cortex (PFC) region, **b** statistical analysis of PSD thickness, **c** statistical analysis of the width of synaptic cleft, **d** statistical analysis of the curvature of synaptic interface in the PFC, **e** protein expression levels of BDNF and PSD-95 in PFC. Data are presented as mean ± SEM. *n* = 3 per group. ^*^P < 0.05, vs lab chow diet group (LC); ^#^*P* < 0.05, vs high-fat diet group (HF). HFL, *L. edodes* β-glucan supplementation in HF diet group

Microglial activation and neuroinflammation are the leading risk factors for cognitive decline, which are involved in the pathogenesis of neurodegenerative diseases via altering synaptic ultrastructure and plasticity [7, 34]. This study examined if *L. edodes* altered synaptic ultrastructure and the expression of synapse-associated markers. Synaptic ultrastructure in the CA1 region of the hippocampus and PFC was analyzed using Transmission electron microscopy (TEM). In the CA1 region of the hippocampus, the decreased thickness of the postsynaptic densities (PSD), the broader of the synaptic cleft and reduced curvature of the synaptic interface were found in HF diet mice (all *P* < 0.05, Fig. 6a–d). Interestingly, *L. edodes* β-glucan administration prevented these pathogenic change induced by the HF diet, exhibiting a thicker PSD, narrower synaptic cleft, and higher curvature of the synaptic interface (all *P* < 0.05, Fig. 6a–d). Moreover, the protein levels of synapse plasticity markers, brain-derived neurotrophic factor (BDNF) and postsynaptic density-95 (PSD-95) were significantly inhibited by HF diet (*P* < 0.05, Fig. 6e); however, their expression levels were recovered after *L. edodes* β-glucan (*P* < 0.05, Fig. 6e). Furthermore, compared to the HF group, a similar trend has been gained in the PFC of mice after *L. edodes* β-glucan supplementation (Fig. 7). In summary, these results indicated that *L. edodes* β-glucan could improve synaptic morphology and plasticity in the hippocampus and PFC, thereby preventing cognitive decline induced by HF diet.

## Discussion

The present study, with an obese cognitive impairment mice model, demonstrated the beneficial effects of *L*. *edodes* β-glucan supplementation on the gut microbiota-brain axis and improvement of cognitive decline. We showed that short-term and long-term *L*. *edodes* β-glucan supplementation alleviated the gut microbial dysbiosis induced by the HF diet. Notably, long-term supplementation with *L*. *edodes* β-glucan significantly improved the cognitive impairment in HF diet-fed mice, which was supported by the inhibition of microgliosis, alleviation of neuroinflammation and improvement of synaptic ultrastructure. Furthermore, long-term *L*. *edodes* β-glucan supplementation significantly mitigated the impairment of colonic barrier and inflammation in HF diet-fed mice. Collectively, these data demonstrated that *L*. *edodes* β-glucan ameliorated the cognitive deficits induced by chronic HF diet, and these neuroprotective effects potentially occurred through the improvement of the colon-microbiota-brain axis. Previously, edible mushrooms have shown beneficial effects on cognition in a cross-sectional study [20]. Here we reported that the main ingredient of edible mushroom, *L*. *edodes* β-glucan, improved the gut microbiota-brain axis. Therefore, the beneficial effects of β-glucan may contribute to the ability of mushroom in the improvement of cognitive function, as described previously in the human study [20].

The gut microbiome has emerged as a major contributor to cognitive health, and it can be remodeled by dietary factors. Recently, our laboratory demonstrated that gut microbiota dysfunction negatively impacts the cognitive impairments induced by HF diet [15, 16, 35]. Nevertheless, only a few studies focused on the alternation of gut microbiota occur before the onset of body weight gain in this model [35]. In previous long-term studies for HF diet for 8–22 weeks, it is reported that the proportion of gut bacteria belong to phylum Bacteroidetes is decreased, while phylum Firmicutes is increased in rodents with an increased ratio of Firmicutes to Bacteroidetes [36]. The alternation of these indexes was also observed in the present study, in which the mice were fed by HF diet for 15 weeks. Moreover, the changes were reversed by the long-term supplementation of *L*. *edodes* β-glucan. Interestingly, in our short-term HF diet for 7 days, although there was a slight increase in the Firmicutes and decrease in the Bacteroidetes, the alteration of these bacteria did not a reach statically significant difference in the gut microbiota of mice. It suggests that microbiota alteration in the Bacteroidetes and Firmicutes is in progress along with the duration of HF diet feeding. However, in the present study, we found that the mean proportion of Proteobacteria and Actinobacteria at phylum was significantly decreased by the short-term HF diet, suggesting the composition of Proteobacteria and Actinobacteria altered early before the significant change in the Bacteroidetes and Firmicutes. In addition, in the long-term study of HF diet, the richness and diversity of gut microbiota in mice were altered [3], characterized by decreased Chao index and Shannon index. While in the present short-term study, HF diet feeding did significantly change the richness (Chao index) and diversity (Shannon index). This indicates that the shift in the composition of gut microbiota, especially in Proteobacteria and Actinobacteria, occurred before any changes in microbiome diversity and richness during HF diet feeding. Importantly, we found that supplementation of dietary *L*. *edodes* β-glucan prevented this shift in microbiota composition, as it significantly increased Bacteroides and decreased Firmicutes. Consistent with our findings, two previous studies have shown a similar capability of *L*. *edodes* β-glucan to alleviate the gut microbiome dysbiosis in aged mice and insulin deficient type 2 diabetic rats [18, 19]*.* In addition, it has been reported that microbiota belonging to the phylum Bacteroidetes is associated with cognition and neurodegenerative diseases [37]. For example, in a cross-section study, a lower abundance of Bacteroides at the genus is reported in the gut microbiota of dementia patients [38]. Interestingly, the consumption of mushrooms has been shown to reduce the risk of mild cognitive impairment in aged individuals [20]. This study found that the increased abundance of Bacteriodetes phylum following *L*. *edodes* β-glucan administration. Thus, it is rationally proposed that dietary *L. edodes* β-glucan in promoting the abundance of certain members of the bacterial community belongs to the Bacteroidetes phylum, contribute to ameliorate the cognitive impairments induced by HF diet.

Emerging research is revealing that gut microbiota has potent effects on gut permeability and endotoxemia [27]. The gut barrier consists of semi-permeable mucosal, as well as epithelial cell layers reinforced by tight junction proteins. This barrier serves to regulate nutrient and water entry and prevents the entry of harmful compounds into extra-luminal tissues. HF diet consumption impairs gut permeability, which, in turn, allows for the influx of adverse substances [39]. A compromised gut barrier makes the intestinal tract potentially vulnerable to the gram-negative bacteria-derived LPS, which upon excess entry into circulation, promotes endotoxemia and systemic inflammation [16, 40, 41]. According to previous studies, we found that HF diet intake dramatically increased intestinal inflammation and diminished intestinal barrier integrity, which is consistent with the increased level of LPS in the sera of mice. However, long-term *L*. *edodes* β-glucan supplementation increased colonic mucus thickness, upregulated colonic tight-junction protein occludin levels and lowered the LPS level in sera, indicating that *L*. *Edodes* alleviated the loss of intestinal barrier integrity induced by HF diet. It is reported that the outer membrane protein of Bacteroidetes can bind to β-glucan [42]. Moreover, the genome of Bacteroidetes encodes many β-glucan lyases and glycoside hydrolases, which are largely involved in the acquisition and metabolism of β-glucan [43]. Thus, it is possible that *L*. *edodes* β-glucan favoured the growth of the β-glucan-degrading Bacteroidetes and its next taxonomic levels observed in our study. In the present study, we found that at the phylum level, the short-term supplementation of *L. edodes* β-glucan for 7 days significantly increased the proportion of Bacteroidetes in mice fed with HF diet. Previously, in a chronic study, the effect of barley β-glucan supplementation for 5 weeks increased Bacteroidetes in gut microbiota in humans [44]. These results suggest that the changes in microbiota, such as Bacteroidetes by the short-term supplementation of β-glucan may maintain after long-term supplementation. In addition, Bacteroidetes has been reported to benefit their host mucus and gut barrier [10]. Therefore, *L*. *edodes* β-glucan might be fermented by Bacteroidetes, to provide an energy source for bacteria within the Bacteroidetes phylum. In accordance with this, the glycan production in mucus was significantly increased by *L*. *edodes* β-glucan supplementation, which thus prevented the epithelial damage induced by the HF diet. This, in turn, might relieve the translocation of bacterial LPS into the blood circulation.

The hippocampus and the cortex, regions implicated in cognitive processing, learning and memory, are particularly vulnerable to inflammation in obesity [32, 45]. It is reported that LPS from the intestinal tract was increased in the cortex and hippocampus of AD patients [46], which suggests that the increased gut permeability and hyperndotoxinemia could contribute to cognition decline. Our results showed that *L*. *edodes* β-glucan administration enhanced the intestinal barrier and resulted in a profound reduction in endotoxinemia, which may contribute to the improvements in cognition we observed by a comprehensive array of behavioral, learning and memory tests. Neuroinflammation is considered to be the link between gut dysbiosis to synaptic and cognitive decline, while it is also one of the key mechanisms underlying various neurodegenerative diseases [47]. LPS over-exposure by intraperitoneal injection has been reported to induce microglial activation and increased expression of proinflammatory cytokines in the brains of mice 48. Moreover, the gut microbiota directly stimulates the production of the proinflammatory cytokines IL-1β and TNF-α [49], which have been shown to impair hippocampal-dependent memories in rodents [50, 51]. In the present study, we found that a long-term HF diet upregulated TNF-α, IL-6 and IL-1β in the PFC and hippocampus, which were attenuated by long-term *L*. *edodes* β-glucan supplementation, indicative of an anti-neuroinflammatory effect of *L*. *edodes* β-glucan.

There is accumulating evidence demonstrates the microglia play a vital role in mediating the cognitive dysfunction in neurodegenerative dysfunctions [52]. Microglia, the resident immune cells of the central nervous system, maintain brain homeostasis and contribute to brain development. However, excessive microglial activation can damage the surrounding healthy neural tissue, and the factors secreted by the dead or dying neurons, in turn, exacerbate the chronic activation of microglia, causing progressive loss of neurons and then cognitive impairment [8, 52]. This study showed the HF diet promoted the accumulation of microglia in the PFC and hippocampus, which was inhibited by long-term *L*. *edodes* β-glucan supplementation. Synaptic structure and plasticity are closely correlated with learning and memory functions [34]. The dysregulation of synaptic formation and plasticity in the hippocampus has been implicated in patients with cognitive impairment and AD [53]. We herein showed the long-term HF diet disrupted the ultrastructural synaptic architecture in the PFC and hippocampus, which was characterized by decreased PSD thickness and broadened synaptic cleft observed by the TEM technique. Notably, long-term *L*. *edodes* β-glucan supplementation prevented the damage of ultrastructural synaptic architecture induced by the HF diet. Consistently, we also found that *L*. *edodes* β-glucan supplementation reversed HF diet-associated decreases in the molecular markers of synaptic plasticity, BDNF and PSD-95 in the PFC and hippocampus. Therefore, *L*. *edodes* β-glucan supplementation significantly improved the ultrastructure and increased synaptic protein expression, which thus supported the enhancement and maintenance in cognitive function despite chronic HF diet feeding following *L*. *edodes* β-glucan treatment.

## Conclusion

In summary, the present study has demonstrated that dietary *L*. *edodes* β-glucan supplementation prevented cognitive impairment induced by the HF diet in mice, which might result from the improvement of the colon-brain axis. The finding provides evidence that by *L*. *edodes* β-glucan can be a novel nutritional treatment to prevent cognitive deficits induced by long-term intake of the western diet.

## Supplementary Information

**Additional file 1: Figure S1.** Long-term *L. edodes *derived β-glucan supplementation prevented the gut microbiota dysbiosis induced by HF diet. (A) the relative abundance of Firmicutes, (B) the relative abundance of Bacteroidetes, (C) the ratio of Firmicutes (FM) to Bacteroidetes (BO), (D) the relative abundance of Proteobacteria, (E) the relative abundance of Actinobacteria, (F) the Shannon index. Data are presented as mean ± standard error of means (SEM). n=5-6 per group. *^*^P<0.05, ^***^P<0.001, vs lab chow diet group (LC); ^#^*P*<0.05, ^###^*P*<0.001, vs high-fat diet group (HF). HFL, *L. edodes* β-glucan supplementation in HF diet group.

**Additional file 2: Figure S2.** Long-term *L. edodes *derived β-glucan supplementation prevented HF diet-induced obesity in mice. (A) cumulative body weight gain, (B) cumulative energy intake, (C) final body weight, (D) liver mass, (E) the mass of subcutaneous, epididymal and brown fats, (F) blood glucose levels during glucose tolerance test (GTT), (G) area under the curve for GTT. Data are presented as mean ± SEM. n=10 per group. ^**^ *P*<0.01, ^***^P<0.001, vs lab chow diet group (LC); ^#^*P*<0.05, ^##^*P*<0.01, ^###^*P*<0.001, vs high-fat diet group (HF). HFL, L. edodes β-glucan supplementation in HF diet group.

## Data Availability

All datasets generated for this study are included in the article additional materials.
