# Supplementary figures and images for: β-Glucan from Lentinula edodes prevents cognitive impairments in high-fat diet-induced obese mice: involvement of colon-brain axis
Source: J Transl Med. 2021 Feb 4;19:54. doi: 10.1186/s12967-021-02724-6 (PMC7863530; doi:10.1186/s12967-021-02724-6)

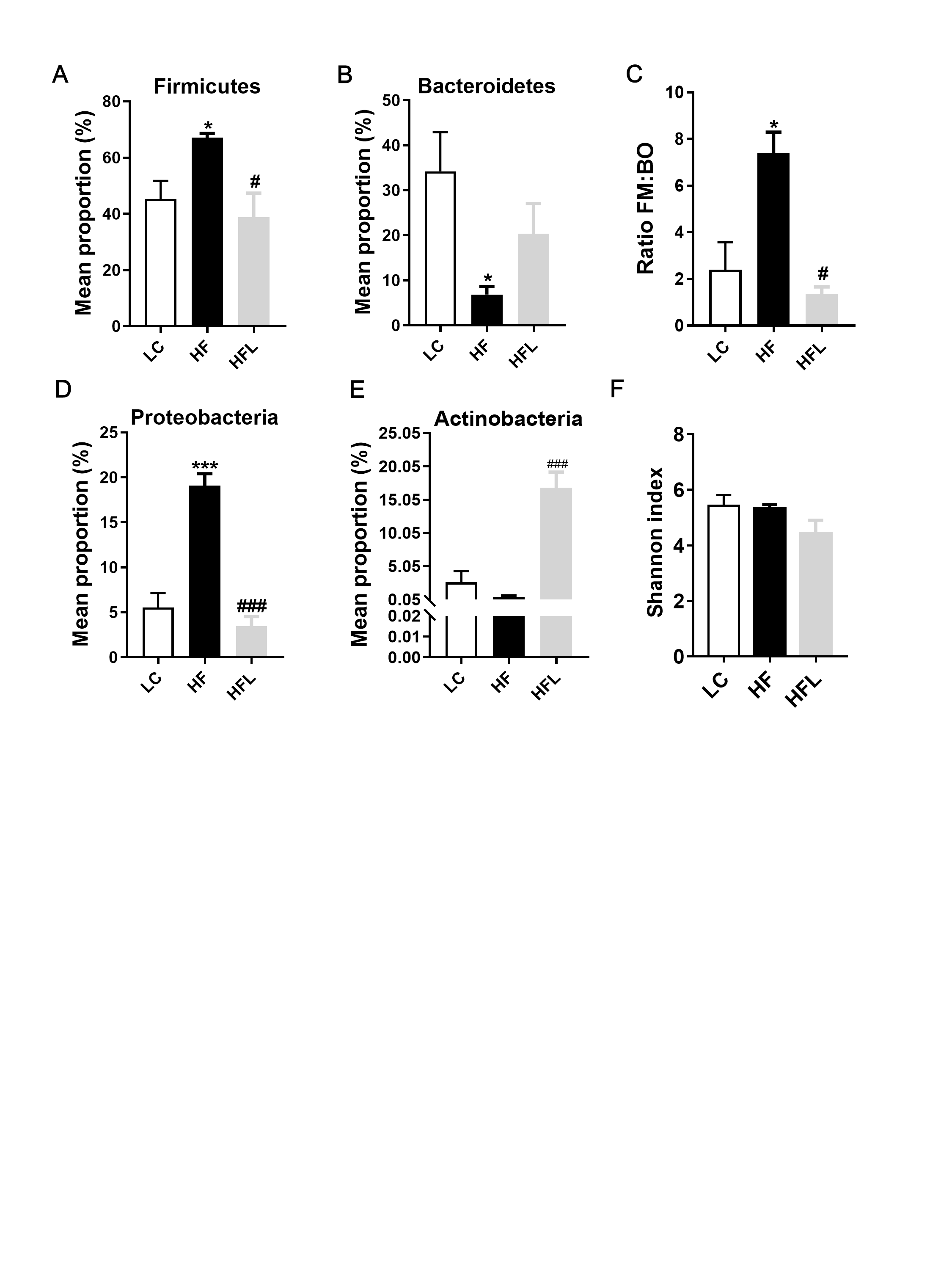

Supplement: Supplementary file 1 — Additional file 1: Figure S1. Long-term L. edodes derived β-glucan supplementation prevented the gut microbiota dysbiosis induced by HF diet. (A) the relative abundance of Firmicutes, (B) the relative abundance of Bacteroidetes, (C) the ratio of Firmicutes (FM) to Bacteroidetes (BO), (D) the relative abundance of Proteobacteria, (E) the relative abundance of Actinobacteria, (F) the Shannon index. Data are presented as mean ± standard error of means (SEM). n=5-6 per group. **P<0.05, ***P<0.001, vs lab chow diet group (LC); #P<0.05, ###P<0.001, vs high-fat diet group (HF). HFL, L. edodes β-glucan supplementation in HF diet group. [file 12967_2021_2724_MOESM1_ESM.tif]

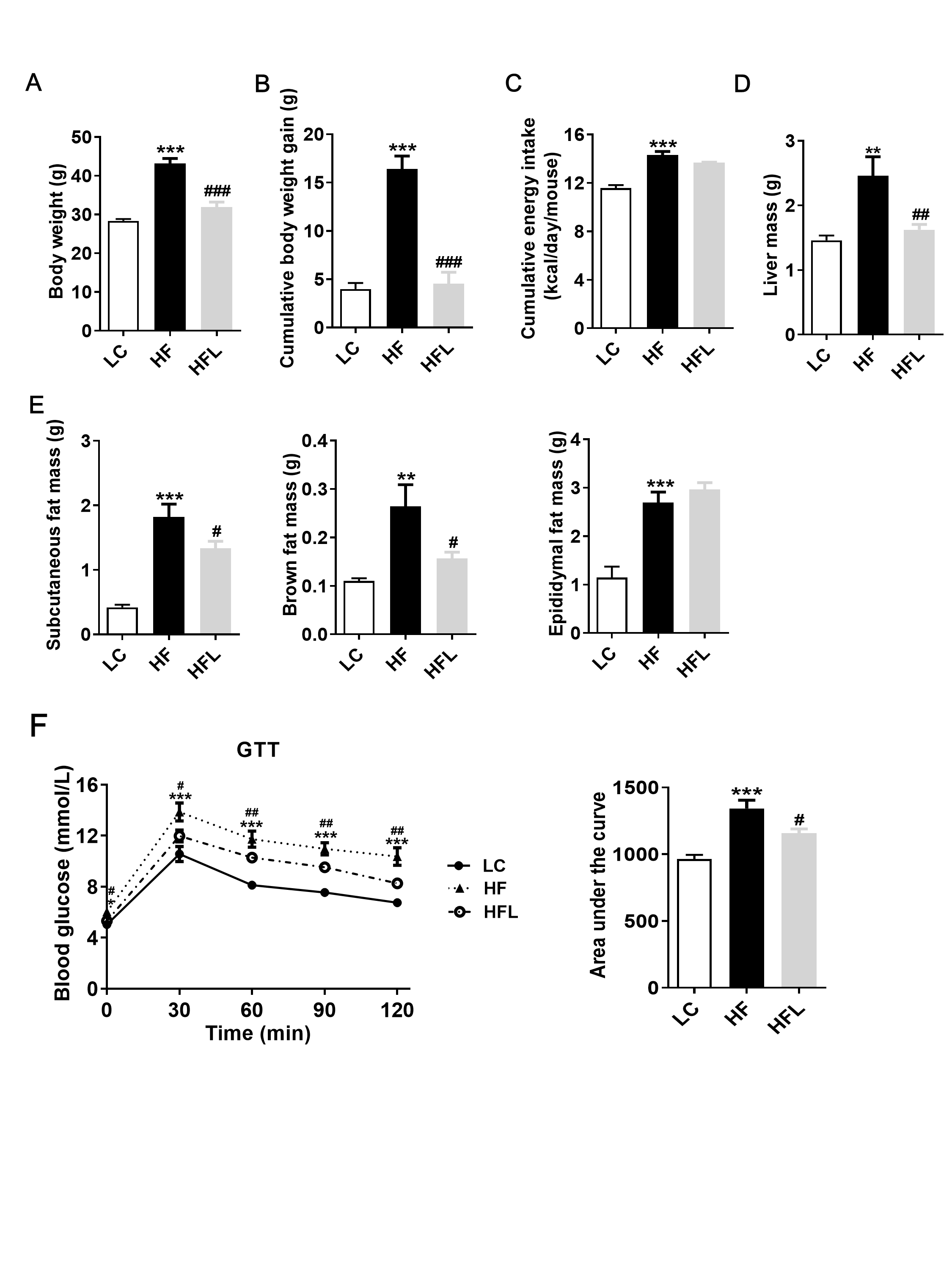

Supplement: Supplementary file 2 — Additional file 2: Figure S2. Long-term L. edodes derived β-glucan supplementation prevented HF diet-induced obesity in mice. (A) cumulative body weight gain, (B) cumulative energy intake, (C) final body weight, (D) liver mass, (E) the mass of subcutaneous, epididymal and brown fats, (F) blood glucose levels during glucose tolerance test (GTT), (G) area under the curve for GTT. Data are presented as mean ± SEM. n=10 per group. ** P<0.01, ***P<0.001, vs lab chow diet group (LC); #P<0.05, ##P<0.01, ###P<0.001, vs high-fat diet group (HF). HFL, L. edodes β-glucan supplementation in HF diet group. [file 12967_2021_2724_MOESM2_ESM.tif]
